# Negative attributions towards people with substance use disorders in South Africa: Variation across substances and by gender

**DOI:** 10.1186/1471-244X-12-101

**Published:** 2012-08-07

**Authors:** Katherine Sorsdahl, Dan J Stein, Bronwyn Myers

**Affiliations:** 1 Department of Psychiatry and Mental Health, University of Cape Town J-Block Groote Schuur Hospital Observatory, Cape Town, South Africa; 2 Alcohol and Drug Abuse Research Unit, Medical Research Council, Cape Town, South Africa

**Keywords:** Alcohol, Drugs, Stigma, South Africa, Gender

## Abstract

**Background:**

Little research has examined attitudes towards people who use substances in low and middle income countries (LMIC). Therefore, the present study examined the attributions made by the general South African population about people who use substances and whether these attributions differ by the type of substance being used, the gender of the person using the substance, or the characteristics of the person making the attribution.

**Method:**

A convenience sample of 868 members of the general public was obtained through street-intercept methods. One of 8 vignettes portraying alcohol, cannabis, methamphetamine or heroin, with either a male or female as the protagonist was presented to each respondent. Respondents’ attitudes towards the specific cases were investigated.

**Results:**

Respondents held equally negative views of the presented substances, with the exception of the cannabis vignette which was considered significantly less “dangerous” than the alcohol vignette. Respondents were more likely to offer “help” to women who use alcohol, but more likely to suggest “coercion into treatment” for men. Individuals who scored higher on the ASSIST were more likely to hold negative attitudes towards substance users and black African respondents were more likely to offer help to individuals who use substances.

**Conclusion:**

The stigma associated with substance use in South Africa is high and not necessarily dependent on the drug of choice. However, a range of factors, including gender of the substance user, and ethnicity of the rater, may impact on stigma. Interventions designed to strengthen mental health literacy and gender-focused anti-stigma campaigns may have the potential to increase treatment seeking behaviour.

## Background

Substance use represents a major public health problem, both globally and in South Africa. Results from the South African Stress and Health Study (SASH), the first nationally-representative study of psychiatric morbidity in South Africa indicate a high lifetime prevalence (13.3 %) and early onset (21 years) of substance use disorders [1]. Despite this high prevalence, only 27.6 % of South Africans who met the criteria for a substance use disorder received treatment in the year preceding the interview [2].

A few studies have attempted to investigate barriers to accessing treatment for substance use. For example, a lack of available services or structural barriers [3] and a low perceived need for treatment [4] are often cited as significant barriers to accessing substance abuse services. Previous research has also identified stigma towards people who use drugs as a barrier to treatment entry, with individuals who need help tending to deny or hide their condition for fear of being negatively labelled [5].

Substance use disorders are highly stigmatised, with several studies reporting that these disorders are more stigmatised than other physical [6-8] and mental disorders [9-12]. For example, a cross-national study conducted by the World Health Organization (WHO) that examined 18 of the most stigmatised conditions (e.g., being a criminal, HIV positive, or homeless), ranked alcohol addiction as the fourth most stigmatised condition, while drug addiction was ranked as the most stigmatised condition [13]. Additionally, although persons with psychotic disorders are often viewed as dangerous and unpredictable, substance use disorders appear to elicit more stigmatising reactions than schizophrenia [14,15]. For example, drug addiction was rated higher than 6 other mental disorders on dimensions of dangerousness to others, being difficult to talk to, and unpredictability [14].

Although there is some research available examining attitudes and attributions towards people who use varying classes of drugs [16-19], none have been conducted in a low and middle income country (LMIC). While studies conducted in high income countries generally found that the use of “harder” drugs such as cocaine was rated more negatively than “softer” drugs such as alcohol or cannabis, it is unclear whether these findings are relevant or can be extrapolated to a LMIC such as South Africa where the profile of drug use is very different and where problematic drinking is one of the single greatest threats to public health [20].

Although a few South African studies have investigated the stigma associated with alcohol use compared to other mental disorders [10,11], there are no data available on the stigma associated with illicit drug use In addition, these previous studies did not examine whether the attributions made about people who used substances varied according to the type of substance being used. A better understanding of the type of attributions made towards South Africans who use substances may be valuable for the development of interventions to reduce stigma and thereby address an important barrier to substance abuse treatment use in the country [5].

Another limitation of previous South African research is the failure to examine whether publicly-held attitudes towards women who use substances differ from those held towards men. As several studies have found that women who use drugs experience high levels of perceived and enacted stigma [21], it is quite likely that attitudes towards women who use drugs are more stigmatizing than those towards men. If this is the case, this could partially explain why women with substance use disorders from disadvantaged communities in South Africa do not access treatment services as readily as men [22]. However, as previous studies have not examined differences in attitudes towards substance abusing men and women, this explanation remains unsupported by research. This lack of data impedes the development of targeted interventions to address this potentially important barrier to substance abuse treatment entry for women.

The present study is a response to these gaps in earlier research on stigma and substance use. Specifically, this paper aims to examine attributions made by the general South African population about people who use substances and whether these attributions differ by the type of substance being used, the gender of the person using the substance, or the characteristics of the person making the attribution.

## Method

### Participants

Participants were members of the general South African public. A convenience sample of 868 participants was obtained through street-intercept survey procedures. Two regions (the Northern and Central suburbs) in the Cape Town metropole were chosen for recruitment so as to ensure a sample that depicts a broad spread of responses. The only inclusion criterion for participation was that respondents had to be over 18 years of age.

### Procedure

In each location, men and women aged 18 and older were randomly approached while in public areas (such as the train station, busy street junctions, and shopping malls) and asked to complete a brief anonymous questionnaire. The fieldworkers described the study and the consent process using a script developed specifically for this study to ensure all participants were approached in a similar manner. Potential participants were informed about the confidentiality and anonymity of the process. Participation was voluntary, as was withdrawal from the study. Consent was then obtained for participation in the study. These questionnaires were administered by teams of fieldworkers who were trained in research methods and the study protocol. The study was approved by the University of Cape Town’s Health Research Ethics Committee.

After recruitment, participants were randomly given one of eight vignettes (case studies) that they were asked to think about while answering the questionnaire. These vignettes referred to either a man or a woman who used alcohol; cannabis (locally referred to as dagga); methamphetamine (locally referred to as tik); or heroin (also referred to locally as unga). The vignettes had enough detail to suggest that each of the characters had developed substance use problems. For example, the vignette depicting alcohol abuse was described using the following: *“Jeremy started drinking when he was a student. He was the life and soul of many parties. By the time he had graduated and got married he was drinking on a daily basis. Although his wife insisted that he drank too much, Jeremy argued that he remained in control. But his work and appearance got worse to the point that his supervisor began to suspect that he might be drinking on the job. A few months later he was involved in a serious car accident, where he wrote off two cars. The police who arrived at the scene of the accident took his blood for alcohol analysis. As his alcohol level was much higher than the legal limit he was charged with drunk driving”.* These vignettes were designed to elicit the participant’s attributions towards the focal substance. These substances were chosen as the focus for the vignettes because their use is highly prevalent in the Cape Town metropole where recruitment occurred.

### Measures

In addition to a number of socio-demographic variables (such as gender, age, education, employment, race and marital status), and questions pertaining to whether or not the participant had ever received treatment for substance use or knew of someone with substance use problems, the following scales were included in the questionnaire that was administered after reading the vignette:

#### Attribution Questionnaire Short Form (AQ-9)

This 9-item scale examines nine stereotypes about people with mental illness (including substance use disorders). These stereotypes include blame, anger, pity, help, dangerousness, fear, avoidance, segregation, and coercion. Item-responses are coded on 9-point opinion scales (ranging from “not at all” to “very much”)[23]. This scale has been previously utilized in a South African population [10].

#### Mental Health Literacy

In addition, respondents were asked to indicate on a three point scale whether they thought the behaviour described in the vignette was a normal response, typical of a weak character, typical of a mental illness, and/or typical of a general medical problem. Response categories for these four mental health literacy items were “Yes”, “Maybe”, or “No”. Participants were able to endorse more than one of the mental health literacy items since it is possible to hold many (often contradictory) explanatory models of mental disorders. Although this has limitations, these questions have been used previously to examine mental health literacy among community samples [10] and samples of people living with HIV [11].

#### Substance use

The Alcohol, Smoking, and Substance Involvement Screening Test (ASSIST; WHO ASSIST Working Group, 2002) was administered to assess the extent of problematic substance use among participants in the study. Although the ASSIST was originally developed to detect and manage substance use and related problems in primary and general medical care settings, it has been used in non-clinical populations to assess risk for substance use disorders (for example see [24]). Based on their scores, participants were categorised into low, moderate or high risk for substance-related problems. Low risk indicates that the participant is at low risk for health and other problems from their current pattern of substance use (with scores of 0–10 for alcohol and 0–3 for illicit drugs). Moderate risk indicates that the participants are at risk for health and other problems from their current pattern of substance use (with scores ranging between 11–26 for alcohol and 4–26 for illicit drugs). High risk indicates that the participant is at high risk of experiencing severe problems (health, social, financial, legal, or relationship difficulties) as a result of their current pattern of use and are likely to be dependent (with scores >26).

### Analysis

Data were analyzed with the Statistical Package for the Social Sciences, version 20.0. The sample was described and differences in the mental health literacy questions were examined using chi-square calculations. The distribution of the vignette scores was tested for normality using the Shapiro-Wilk test (and found to be normally distributed). Analysis of variance procedures were performed to identify differences between the respondents’ attributions toward the various substances, followed by multiple post-hoc comparisons using the Scheffé test. For each of the four substances examined in this study, independent samples *t*-tests were then conducted to compare whether the scores obtained for the nine attribution stereotypes differed according to the gender of the character portrayed in the vignette. Finally, nine multivariate linear regression models were developed to establish the adjusted associations between the demographic and substance use- related variables of the respondent, the various classes of substances considered by each vignette, and the 9 AQ stereotypes (entered as dependent variables).

## Results

### Sample characteristics

Details of the demographic characteristics of the sample are provided in Table 1. Most participants were single (81 %), unemployed (57 %), and did not complete high school (55 %). In terms of ethnicity, the majority of respondents were black African (56 %) followed by Coloured (30 %). The terms “White, Black African, Asian/Indian, and Coloured” refer to demographic markers that were chosen for their historical significance and are still used in South Africa today. Coloured refers to a grouping of people of mixed race ancestry that self-identify as a particular ethnic and cultural grouping in South Africa. The continued use of these markers is important for identifying continuing disparities in services and monitoring improvements in health and socio-economic disparities in South Africa.

**Table 1:** Demographic and substance use characteristics of the sample

**Demographic and substance use characteristics**	**Total Sample (%, n)**	**Female (n = 443) (%, n)**	**Male (n = 405) (%, n)**	**p-value**
**Age** (M, SD)	27 (6.4)	26 (6.4)	27 (6.5)	<0.01*
**Race**				0.92
Black	56 (457)	55 (233)	56 (221)	
White/Asian	13 (104)	13 (55)	13 (49)	
Coloured	30 (259)	32 (136)	31 (122)	
**Marital Status**				0.02*
Single/widowed/Divorced	81 (704)	78 (344)	84 (341)	
Married/Cohabitating	19 (164)	22 (99)	16 (64)	
**Education**				0.15
Not completed high school	55 (468)	52 (229)	57 (229)	
Completed high school	45 (388)	48 (209)	43 (171)	
**Employment (yes)**	43 (353)	59 (255)	45 (176)	0.21
**Substance Use ASSIST score** (M, SD)	17 (13.8)	16 (14.1)	19 (13.4)	<0.01*
**Received Treatment for substance use** (Yes)	8 (64)	6 (26)	9 (38)	0.05*
**Know someone with substance use problem** (Yes)	59 (485)	59 (253)	58 (230)	0.89

Eight percent of participants had received previous treatment for substance use and more than half (59 %) knew someone with a substance use problem. The mean score on the ASSIST was 17 indicating that, on average, respondents were at moderate risk for a substance use problem. A significantly greater proportion of male compared to female respondents had received prior treatment for substance use problems and men were significantly more likely to have higher scores on the ASSIST than women.

### Mental health literacy

Overall, only 53 % of the respondents reported that the case study presented to them was typical of a mental illness. Additionally, 34 % believed the descriptions were a “normal response,” and 43 % believed the behaviours were typical of a general medical condition. More than half (64 %) reported that the behaviour described in the vignettes was typical of a weak character. This pattern of results remained consistent, regardless of the class of substance being considered by the vignette (Table 2).

**Table 2:** Mental Health Literacy: beliefs about substance use behaviour portrayed in the vignettes

**Believe that behaviour is...**	**Total (n = 868) % yes**	**Alcohol (n = 213) % yes**	**Cannabis (n = 229) % yes**	**Methamphetamine (n = 217) % yes**	**Heroin (n = 209) % yes**	**p-value**
Normal response	34	38	33	28	37	0.21
Typical of weak character	64	67	65	61	65	0.71
Typical of mental illness	53	50	54	57	51	0.31
Typical of general medical problem	43	53	44	38	55	0.26

### Negative attributions towards people with substance use disorders

Across all four vignettes, the mean score for each of the nine AQ-9 items was above the neutral score of 4.5. Subsequent analyses of variance for the nine items found that perceptions of “dangerousness” differed according to the type of substance being used (p < 0.001; Table 3). Post-hoc analysis found that the vignette depicting a cannabis user was considered significantly less “dangerous” than the character in the alcohol vignette (p < 0.001). No other differences were found (see Table 3).

**Table 3:** Comparison of the AQ-9 item scores across the various classes of substances

**AQ-9 items**	**All Vignettes (n = 868)**		**Alcohol (n = 213)**		**Cannabis (n = 229)**		**Methamphetamine (n = 217)**		**Heroin (n = 209)**		**Comparison of Means *p***
	**Mean**	**SD**	**Mean**	**SD**	**Mean**	**SD**	**Mean**	**SD**	**Mean**	**SD**	
Pity	5.3	2.4	5.1	2.6	5.4	2.3	5.4	2.3	5.3	2.2	0.51
Dangerousness	6.2	2.1	6.5	2.1	6.0	2.2	6.0	2.2	6.1	2.0	0.04*
Fear	5.5	2.4	5.5	2.5	5.3	2.5	5.3	2.5	5.5	2.2	0.45
Blame	6.0	2.3	6.2	2.2	6.0	2.1	6.0	2.1	6.0	2.3	0.48
Coercion	5.9	2.4	6.2	2.4	5.9	2.3	5.9	2.3	5.7	2.4	0.23
Anger	5.5	2.5	5.7	2.4	5.6	2.4	5.6	2.4	5.4	2.3	0.13
Help	5.7	2.3	5.9	2.5	5.7	2.4	5.6	2.4	5.5	2.2	0.41
Segregation	5.4	2.5	5.2	2.6	5.3	2.5	5.3	2.5	5.6	2.3	0.45
Avoidance	4.8	2.5	4.5	2.6	4.6	2.5	4.6	2.5	5.1	2.4	0.08
Total Score	5.6	1.2	5.6	1.3	5.5	1.2	5.5	1.2	5.6	1.0	0.80

Only a few significant differences were found when comparing scores on the AQ-9 items by the gender of the character portrayed in the vignette (Table 4). For the alcohol vignette, respondents were more likely to offer “help” to a female rather than a male drinker (*p = 0.03),* while “coercion” into treatment was more acceptable for male users *(p = 0.02).* Respondents reported “avoiding” female cannabis users more than male users *(p = 0.02),* and report “coercion” into treatment as an option significantly more for a women who uses methamphetamine compared to a man *(p = 0.004).*

**Table 4:** Substance use Stigma and Gender: A comparison across the various classes of substances

**AQ-9 items**	**Alcohol**			**Cannabis**			**Methamphetamine**			**Heroin**		
	**Female**	**Male**	**p-value**	**Female**	**Male**	**p-value**	**Female**	**Male**	**p-value**	**Female**	**Male**	**p-value**
Pity	5.10 (2.49)	5.02 (2.49)	0.81	5.36 (2.27)	5.46 (2.41)	0.76	5.44 (2.56)	5.13 (2.38)	0.37	5.22 (2.16)	2.29 (0.21)	0.76
Dangerousness	6.71 (2.11)	6.28 (2.08)	0.14	5.96 (2.28)	6.00 (2.15)	0.93	6.26 (2.45)	6.46 (1.92)	0.50	1.96 (0.21)	2.00 (0.19)	0.67
Fear	5.58 (2.60)	5.50 (2.47)	0.80	5.50 (2.45)	5.11 (2.46)	0.24	5.56 (2.36)	5.77 (2.43)	0.54	5.59 (2.08)	5.46 ((2.32)	0.68
Blame	6.04 (2.49)	6.40 (1.97)	0.49	6.17 (2.08)	5.78 (2.15)	0.16	5.91 (2.64)	5.72 (2.41)	0.61	5.88 (2.29)	5.95 (2.32)	0.83
Segregation	5.48 (2.76)	4.93 (2.47)	1.37	5.35 (0.22)	5.21 (0.23)	0.66	5.58 (2.58)	5.22 (2.49)	0.31	5.51 (2.40)	5.63 (2.30)	0.70
Anger	5.97 (2.61)	5.41 (2.40)	0.12	5.76 (2.35)	5.52 (2.37)	0.45	5.11 (2.78)	5.23 (2.49)	0.62	5.51 (2.41)	5.28 (2.22)	0.49
Help	6.29 (2.48)	5.51 (2.46)	0.03*	5.95 (2.30)	5.48 (2.40)	0.14	5.69 (2.32)	5.72 (2.21)	0.92	5.23 (2.31)	2.41 (0.27)	0.34
Avoidance	4.22 (2.67)	4.80 (2.58)	0.12	5.35 (2.26)	5.21 (0.23)	0.02*	5.06 (2.65)	4.67 (2.25)	0.26	5.23 (2.31)	5.00 (2.41)	0.49
Coercion	5.79 (2.36)	6.57 (2.33)	0.02*	4.24 (0.24)	5.01 (0.22)	0.71	6.24 (2.54)	5.55 (2.48)	0.04*	5.57 (2.41)	5.81 (2.27)	0.49

The multivariate linear regression models found several factors that were significantly associated with the AQ-9 stereotypes of “pity”, “dangerousness”, “blame”, “anger”, “help”, “segregation” and “avoidance” (Table 5). There were no factors significantly associated with “fear” and “coercion”.

**Table 5:** Adjusted Associations between Demographic, and Substance Use Related Variables on Attribution Questionnaire Items

**Variables**	**Attribution stereotype**													
	**Pity**		**Dangerous**		**Blame**		**Anger**		**Help**		**Segregation**		**Avoidance**	
	**β**	**p-value**	**β**	**p-value**	**β**	**p-value**	**β**	**p-value**	**β**	**p-value**	**β**	**p-value**	**β**	**p-value**
Gender	0.04	0.33	0.03	0.49	0.04	0.35	0.08	0.03*	−0.01	0.72	−0.02	0.56	0.06	0.09
Age	0.12	0.01*	−0.04	0.54	0.8	0.11	0.05	0.28	−0.10	0.04*	0.02	0.67	0.02	0.62
Education (not completed high school)	−0.67	0.93	0.02	0.45	−0.01	0.93	−0.09	0.02*	0.07	0.46	−0.20	0.62	−0.03	0.40
Employment (no)	−0.10	0.01*	−0.03	0.39	−0.06	0.15	−0.02	0.64	−0.03	0.45	−0.01	0.85	0.03	0.38
Race (black)														
Coloured	0.01	0.75	0.02	0.64	0.06	0.15	−0.02	0.65	−0.10	0.01*	−0.11	0.01	0.03	0.41
White	−0.50	0.22	−0.06	0.17	−0.02	0.66	−0.06	0.13	−0.10	0.01*	−0.20	0.01	0.06	0.14
Marital Status (no)	−0.03	0.51	0.03	0.48	0.04	0.33	0.01	0.93	0.11	0.02*	0.03	0.53	0.03	0.49
Substance Use ASSIST Score	0.20	0.66	−0.04	0.37	0.01	0.78	0.02	0.64	−0.09	0.03*	−0.02	0.54	0.09	0.02*
Received Treatment for substance use (no)	0.00	0.99	−0.01	0.84	0.08	0.03*	0.06	0.11	−0.07	0.08	0.02	0.59	−0.01	0.88
Know someone with substance use problem (no)	0.05	0.20	0.02	0.65	−0.05	0.24	−0.05	0.20	−0.04	0.27	0.04	0.34	0.05	0.21
Vignette (ref alcohol)														
Marijuana Vignette (yes)	0.03	0.51	−0.09	0.04*	−0.06	0.17	0.00	0.99	0.01	0.99	0.01	0.96	−0.01	0.90
Methamphetamine Vignette (yes)	0.02	0.67	0.01	0.93	−0.08	0.10	−0.08	0.07	−0.01	0.89	0.02	0.75	0.02	0.71
Heroin Vignette (yes)	0.03	0.50	−0.04	0.45	−0.05	0.28	−0.06	0.16	−0.04	0.27	0.04	0.36	0.70	0.13

Older respondents were more likely to feel “pity” towards substance users (β = 0.12, p < 0.001) and less likely to offer help (β = −0.10, *p = 0.04)* than younger respondents. Higher educated respondents were less likely to report feelings of “anger” towards people who use substances (β = −0.12, *p = 0.01)* than those with less education. Compared to black African participants, white (β = −0.10, *p = 0.01*) and Coloured (β = −0.10, *p = 0.01)* participants were less likely to offer “help” to someone who uses substances and less likely to agree that segregation would be beneficial for substance users than black African respondents. Moreover, married respondents (β = −0.11, *p = 0.02)* were more likely to want to “help” the character in the vignette.

Respondents who scored higher on the ASSIST were less likely to offer help (β = −0.09, *p = 0.03)* and more likely to report wanting to “avoid” the characters in the vignettes (β = −0.09, *p = 0.02)* than respondents who scored low. Those who had received treatment for substance use previously were more likely to “blame” the individuals in the vignette for their substance use (β = −0.08, *p = 0.03)* than those who had never received treatment. The character in the marijuana vignette was less likely to be viewed as “dangerous” (β = −0.09, *p = 0.04)* than the character in the alcohol vignette.

## Discussion

This study, the first of its kind to examine public stigma towards people who use substances in South Africa, had four important findings. First, high levels of public stigma were reported for all classes of substances investigated in this study. Specifically, the average scores on the AQ-9 for the various classes of substances ranged from 4.8-6.2 (well above the neutral score of 4.5); indicating that people who use substances are viewed negatively by the general population. Furthermore, as these AQ-9 scores are higher than those obtained for depression (4.7), schizophrenia (4.9), panic disorder and PTSD (4.9) by a previous study that used similar methods and was conducted in a similar location [10], these findings suggest that the South African public view people who use substances in a more negative light than persons with other mental disorders. Although it is plausible that between-study factors could have partially accounted for this difference, this finding is in keeping with results from previous studies conducted in other contexts [12,14,15]. One explanation for this difference may lie in attributions of personal culpability that are more often made about people with substance-related problems compared to people with other mental disorders, especially when these mental disorders are viewed as having structural causes [25,26]. These findings suggest that in order to reduce the high levels of stigma towards people who use substances, public mental health literacy programmes need to be expanded to include better information about substance use disorders. For example, accurate information about the neurobiological roots of substance use and other mental disorders may help challenge attributions about personal responsibility for substance use disorders. Given our findings of low levels of mental health literacy relating to substance use disorders, this may be a powerful intervention for reducing stigma towards people who use substances [27].

Second, all the characters in the vignettes were viewed equally negatively by the respondents regardless of the type of substance being used; with the exception of the cannabis user who was considered significantly less “dangerous” than someone who uses alcohol. These findings are inconsistent with previous studies conducted in developed country settings that found the use of “harder” drugs (such as methamphetamine or heroin) to be rated more negatively than the use of “softer” drugs such as alcohol and cannabis [28,29]. One explanation for why respondents may have viewed people with alcohol problems as equally as dangerous as people who use “harder” drugs may lie in the high prevalence of problem drinking in South Africa [1,20] and the large burden that alcohol places on communities in terms of alcohol-related injuries, alcohol-related violence and alcohol-related crime [30].

The third important finding is that publicly held attitudes towards women who used substances differed from those held towards men. Although attributions of pity, dangerous, blame, anger and segregation did not differ between men and women, vignettes of women who used cannabis and methamphetamine evoked more negative attributions from respondents (namely avoidance and coercion in treatment) than vignettes of men who used these substances. This is not altogether surprising given previous research in South Africa and elsewhere which has noted that women with substance use disorders are perceived more negatively than men [5,21]. This may be because women’s drug use is strongly associated with perceived inability to fulfil traditional gender roles, such as taking care of dependent children [31] and also because methamphetamine use, in particular, is strongly associated with discourses of female sexual availability and “immoral” behaviour [5] which goes against widely held beliefs about appropriate (conservative) female sexual conduct in a predominantly patriarchal country [32]. In contrast, the vignettes of women who used alcohol evoked positive responses (of offers to help) compared to those for men, which evoked responses of coercion into treatment. This finding is inconsistent with earlier studies, conducted in developed country settings, which reported high levels of stigma towards women with alcohol-related problems [33]. While the reasons for this unexpected finding are not clear, one potential explanation may lie in public perceptions that women’s drinking is less associated with adverse social consequences (such as crime and violence) than men’s drinking [34]. Nonetheless, this explanation requires further investigation through qualitative research that unpacks the reasons why the public perceives men with alcohol-related problems in a more negative light compared to their female counterparts.

Finally, it appears that people who use substances more frequently held differing attitudes towards people with substance use disorders than those who reported using substances less often. Although attributions of pity, dangerous, blame, anger and segregation did not differ between users and non-users, respondents with higher substance use involvement scores were less likely to offer “help” and more likely to report wanting to “avoid” the characters in the vignettes than respondents with low substance use involvement scores. In a similar vein, respondents who had received previous substance abuse treatment were more likely to “blame” the individuals in the vignette for their substance use than those who had never received treatment. These findings may be explained by previous research examining self-image bias in drug use attributions which found that people who use substances were more likely than non-users to make stable, less controlled and more dispositional attributions about their own use and other’s use of substances [35]. It is argued that substance users often rationalize their own behaviour by adopting an “addicted” explanation of substance use, minimizing personal responsibility for their behaviour [36]. These findings may also reflect community norms which distinguish between people with unproblematic substance use and those who have developed substance use disorders; with the latter being viewed as “mad, “bad”, and having “lost control” [5]. These norms may result in people who use substances wanting to distance themselves from individuals with substance use problems. These possible explanations however require further investigation.

These four findings should be considered in the light of some limitations. First, these data are based on self-report and are therefore subject to the limitations of self-report bias. Second, responses may have been influenced by social desirability, thus leading to an underestimation of stigma levels. Third, because of convenience sampling, the results may not be generalizable to the broader South African population, although we did try to ensure that the sample was broadly representative of the socio-demographic composition of the population in the Western Cape Province where the study occurred. Related to this, although fieldworkers randomly approached people to participate in the study, they may have inadvertently self-selected participants to approach, hence biasing the sample. Finally, the vignettes used to elicit attitudes may provide only a partial picture of the disorder. Reading a description of an individual’s symptoms may not directly relate to one’s ability to recognize symptoms of psychopathology. Similarly, a self-report describes one beliefs about what one would do when confronted with a particular situation, but it does not describe what one actually does in that situation.

## Conclusions

This study revealed high levels of public stigma across all classes of substances, the cannabis user was considered significantly less dangerous than someone who uses alcohol, there were gender differences in attitudes towards people who use substances, and the more an individual used substances the more likely they were to hold a negative attitude. These findings highlight the importance of developing targeted interventions specifically for the South African context. Interventions designed to strengthen mental health literacy and gender-focused anti-stigma campaigns have the potential to increase treatment seeking behaviour.

## Competing interests

The author(s) declare that they have no competing interests.

## Authors’ contributions

BM was involved in conceptualizing the study design and in drafting and reviewing the manuscript. DS was involved in drafting of the manuscript. KS was involved in designing the research, and participated in every aspect of the study from its inception and the production of this manuscript. All authors read and approved the final manuscript.

## Pre-publication history

The pre-publication history for this paper can be accessed here:

http://www.biomedcentral.com/1471-244X/12/101/prepub
